# A High Sensitivity Three-Dimensional-Shape Sensing Patch Prepared by Lithography and Inkjet Printing

**DOI:** 10.3390/s120404172

**Published:** 2012-03-28

**Authors:** Yi-Ren Huang, Sheng-An Kuo, Michal Stach, Chia-Hsing Liu, Kuan-Hsun Liao, Cheng-Yao Lo

**Affiliations:** 1 Institute of NanoEngineering and MicroSystems, National Tsing Hua University, No. 101, Section 2, Kuang Fu Road, Hsin Chu 30013, Taiwan; E-Mails: yrhuangsun@gmail.com (Y.-R.H.); ksa0928@hotmail.com (S.-A.K.); stasekcz@seznam.cz (M.S.); s9935804@m99.nthu.edu.tw (K.-H.L.); 2 Department of Power Mechanical Engineering, National Tsing Hua University, No. 101, Section 2, Kuang Fu Road, Hsin Chu 30013, Taiwan; E-Mail: caspar.rush@gmail.com

**Keywords:** curvature sensor, flexible electronics, inkjet printing

## Abstract

A process combining conventional photolithography and a novel inkjet printing method for the manufacture of high sensitivity three-dimensional-shape (3DS) sensing patches was proposed and demonstrated. The supporting curvature ranges from 1.41 to 6.24 × 10^−2^ mm^−1^ and the sensing patch has a thickness of less than 130 μm and 20 × 20 mm^2^ dimensions. A complete finite element method (FEM) model with simulation results was calculated and performed based on the buckling of columns and the deflection equation. The results show high compatibility of the drop-on-demand (DOD) inkjet printing with photolithography and the interferometer design also supports bi-directional detection of deformation. The 3DS sensing patch can be operated remotely without any power consumption. It provides a novel and alternative option compared with other optical curvature sensors.

## 1. Introduction

Shape monitors are usually realized by deformation sensors using methodologies such as Bragg diffraction gratings [1,2], Moiré patterns [3,4], and laser distance measurements [5,6]. The optical interference takes advantages of interference by two coherent lights from a laser source and the object. With the interfered patterns received by the detector and analyzed by computer-aided algorithms, one understands when the shape of the object changes. However, because of the high sensitivity and very small tolerance window of optical interference, slight environmental variations such as heat flow between the detector and the object, vibrations of the system, and the instability of the light source, any unprotected diffraction method becomes unpredictable and uncontrollable. Similarly, although current mature laser distance measurement methods provide outputs, laser-based detection methodologies require complicated handling on scanning for large area monitoring. Besides the aforementioned potential issues, the scanning part of the laser-based system itself also generates inaccuracies. Other optical fiber based ideas also show shortcomings when sensing a large area.

On the other hand, the Moiré pattern uses at least two periodical patterns to generate special marks which change upon the relative movement of shift of the two patterns. The potential issue of the Moiré pattern methodology is that the two periodical patterns have to be kept within the resolvable range for Moiré pattern generation. Distant separation of the two patterns degrades the resolution. This greatly reduces the detection size of the surface and the sensing range of the curvature of the object. Furthermore, one of the Moiré patterns should be generated on the object, which limits the application of the object and the sensing system.

We previously proposed a curvature sensor [7] with unique characteristics such as distant sensing and zero-power consumption which was based on the microelectromechanical system (MEMS)-controlled Fabry-Pérot color interferometer [8]. The previous work was done partially by roll-to-roll printing techniques which cannot precisely control the printed thickness of the cavity of the interferometer, which in turn led to a specific but limited operation range.

The major improvement of this work focuses on the development of an inkjet drop-on-demand (DOD) printed three-dimensional-shape (3DS) sensor in the form of a patch and its failure and operation limit analysis by the finite element method (FEM). The inkjet printing technique provides flexibility and controllability of droplet size, droplet volume, droplet wetting behavior, and the final thickness of the cavity of the interferometer. A comparison between the conventional photolithography and various printing techniques was done before [9], thus this work focuses on the possibility of replacing the gravure printing by inkjet DOD printing from a cost-efficiency and environmentally-friendly viewpoint. The failure analysis by FEM provides and proves the model for saturation behavior after slight or even severe buckling.

## 2. Modeling and Simulation

### 2.1. General Buckling Model

**Figure 1.**
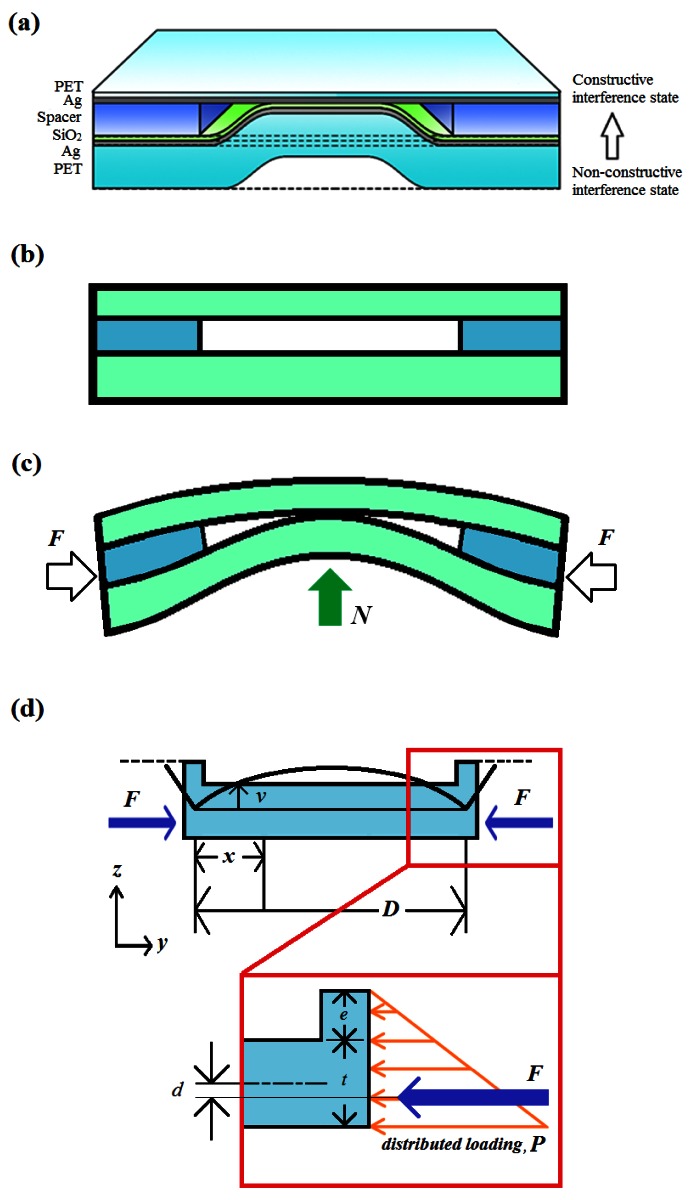
Models and structures of the 3DS sensing patch. (**a**) Layer property and material definition; (**b**) schematic plot for flat state; (**c**) schematic plot for buckling state; and (**d**) buckling model and parameter definition.

The 3DS sensing patch is shown in Figure 1(a) and was modeled based on the theory of buckling of columns [10] for the maximum contact area under either a flat state (Figure 1(b)) or a deformed state of the substrate (Figure 1(c)) with an assumption that there is only one extreme value under bending.

The bending moment based on the theory of buckling of columns for small deformation was defined as:

(1) M=F(d+v)

where *M* is the bending moment, *F* is the axial loading in y-direction, *d* is the eccentric distance between the axial loading (*F*) and the centroid of the structure cross section, and *v* is the vertical displacement under buckling. Other parameters for Figure 1(d) and this work were listed in Table 1. The purpose of this modeling and simulation is to find out the following: (i) Is there any contact for Figure 1(c) under buckling? (ii) If the answer for (i) is YES, what happens after the initial contact? (iii) Following (ii), is there any limitations for the contact?

From Equation (1), the homogeneous solution and the particular solution for the ordinary differential equation were obtained from the differential equation for the deflection curve [11]:

(2) $$EI\frac{d^{2}v}{dx^{2}}=F(d+v)$$

The relationship between *x* and *v* then became:

(3) $$v(x)=C_{1}\sinh(\sqrt{\frac{F}{EI}}x)+C_{2}\cosh(\sqrt{\frac{F}{EI}}x)-d$$

From the model of Figure 1(d), the displacement (*v*) is zero with boundary conditions of *x* = 0 and *x* = *D*. The parameters of *C*_1_ and *C*_2_ in Equation (3) were obtained by differentiating Equation (3):

(4) $$C_{1}=\frac{d[1-\cosh(\sqrt{\frac{F}{EI}}D)]}{\sinh(\sqrt{\frac{F}{EI}}D)}$$

(5) $$C_{2}=d$$

An overall consideration for the vertical displacement thus became:

(6) $$v(x)=d[-\tanh(\sqrt{\frac{F}{EI}}\frac{D}{2})\sinh(\sqrt{\frac{F}{EI}}x)+\cosh(\sqrt{\frac{F}{EI}}x)-1]$$

The maximum vertical displacement (*v*_max_) was derived and happened in the middle of the structure (*x* = *D*/2) with condition *v*′(*x*) = 0. Thus:

(7) $$v_{\max}=d[\mathrm{sech}(\sqrt{\frac{F}{EI}}\frac{D}{2})-1]$$

### 2.2. Layer Contact under Buckling

Equation (7) provided a general solution for both the upper layer and the lower layer shown in Figure 1(c). By individually considering each layer's buckling behavior with Equation (3), maximum displacement of the upper (*v*_max,u_) and the lower (*v*_max,l_) layer could be compared for contact behavior.

**Figure 2.**
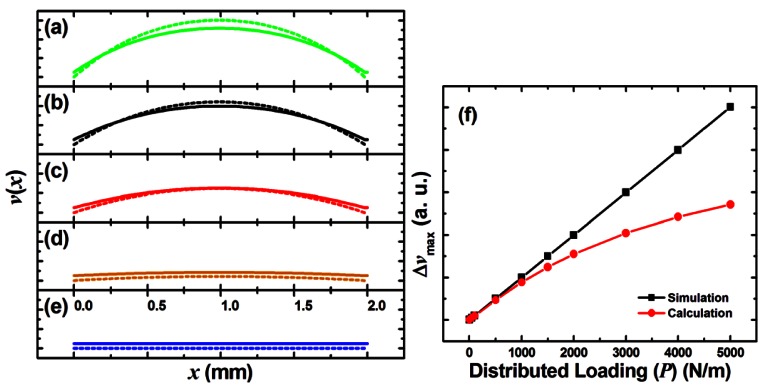
The relationship between location and corresponding displacement under (**a**) P = 2,000, (**b**) P = 1,500, (**c**) P = 750, (**d**) P = 300, and (**e**) P = 1 N/m with the upper layer (solid line) and the lower layer (dashed line) for each graph; (**f**) Distributed loading in real case implied a maximum displacement difference saturation.

Figure 2(a–e) shows the buckling trends of both the upper and the lower layer. Because of the thickness (*t*) difference, the lower layer deformed faster and more than the upper layer under the same axial loading (*F*). Thus the lower layer gradually came closer to the upper layer and started the contact. The results showed that the spacings at both ends of the device were the same but the maximum displacement difference between the two layers gradually reduced. Contact happened only when the difference of displacement maxima was larger than spacer height (*e*):

(8) $$\Delta v_{\max}=v_{\max,1}-v_{\max,\text{u}}\geq e$$

However, the final buckling behavior implied a saturation contact with limits which could not be set as the boundary condition during calculation. The increased displacement difference in turn implied the expansion of the contact area when boundary condition was taken into consideration in a real case: the extra load should be released to both sides from top center and the *v*_max,l_ should also be limited by the *v*_max,u_ throughout the device width (*D*).

Figure 2(f) shows the simulation results performed by ANSYS^®^ (13.0) and the calculation results from Equation (7) with typical values mentioned before. The settings of the simulation were: Element Types/Structure Mass (Solid)/8 node 183. The simulation used a simplified single layer model which did not include the spacer in Figure 1(d). Because the single layer directly reflected the axial loading, the displacement increased linearly. In contrary, the calculation data showed a saturation behavior which reflected the real case that the displacement of a single layer reached its *v*_max_ like a fold. This comparison showed the eligibility of the model and the abundance of its definitions.

### 2.3. Area Expansion after Contact

As mentioned in Section 2.2, the loading pushed the lower layer upwards and finally contacted the upper layer. Because of the boundary limit by the upper layer, the contact area expanded from the top center and gradually reached device edges. The simulation of the contact area change was done with the same settings besides a complete structure, as shown in Figure 1(b), was used. Because the simulation could not perform the horizontal force with vertical deformation, an extra normal force (*N*) was applied to monitor the buckling behavior and contact area change as implied in Figure 1(c).

**Figure 3.**
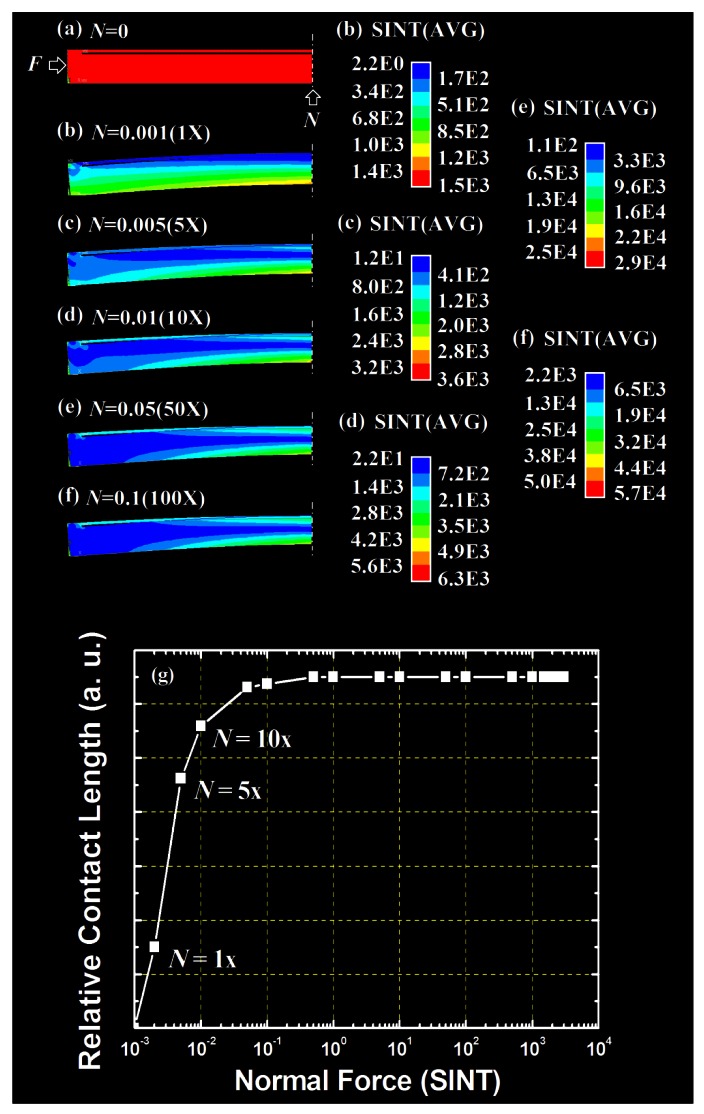
(**a**–**f**) The simulation results with complete structure under different normal force; (**g**) After a specific point, the contact length in (**a**–**e**) saturated.

The two layers deformed individually under small load without contact was shown in Figure 3(a). A starting point of the contact area appeared when the load was sufficient as shown in Figure 3(b–e). After a linear region, a maximum contact area was reached as shown in Figure 3(f). The color legends for simulations were listed accordingly. The same color in the same simulation result showed the same stress intensity (SINT) which also represented the intensity of the normal force (*N*) in Figure 3(g). The simulation results clearly showed the contact saturation behavior after a specific point. The complete structure simulation indicated that the contact area expanded to both sides from top center because of the boundary limit by the top layer.

### 2.4. Area Expansion Limit

As mentioned in Section 2.2, the real case of buckling deviated from its simulation result like a fold. As a result, not only the *v*_max_ was limited but also its contact area reached a maximum. Figure 3(g) shows the behaviors of the contact length which extended from the structure top center towards its edge. This behavior reflected the implication of the *v*_max_ in Figure 2(f) and proved the concept of area expansion after the initial contact.

Figures 2 and 3 left an obvious operation window that under a specific axial loading, the buckling of both layers started until the initial contact happened. Also right after the initial contact happened, the buckling of the lower layer was limited by the buckling of the upper layer which resulted in the contact area expansion towards both sides. Before the contact area reached its maximum, the aforementioned force–displacement and force–length relationships were linear. However, the force–length relationship saturated after the contact area reached its maximum. After the saturation, the contact area would not increase no matter how large the force was applied. This satisfied the buckling model.

## 3. Process

**Figure 4.**
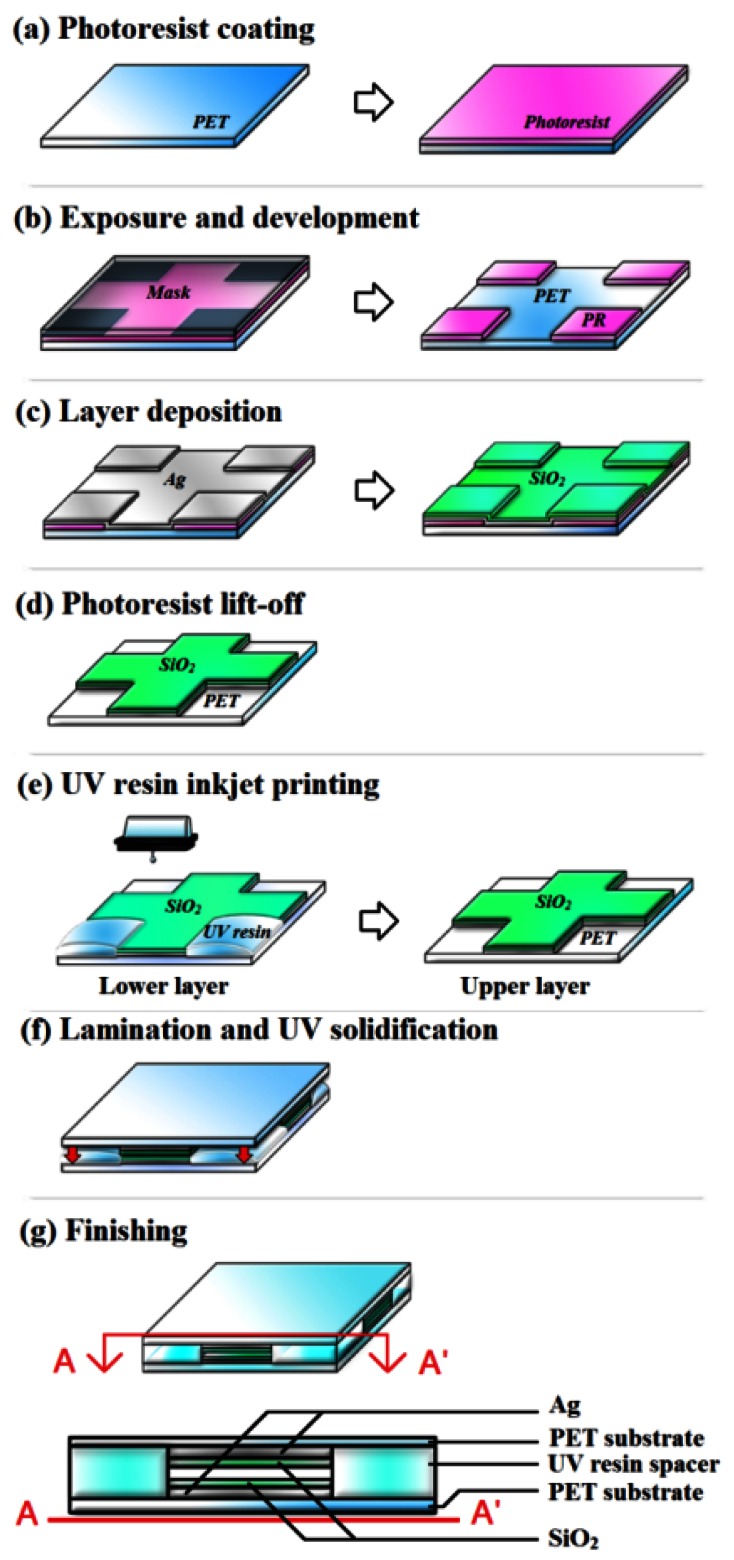
The sequential process steps for the 3DS sensing patch.

Inkjet printing process is believed to be one of the most efficient processes from the economical viewpoint. Inkjet printing is also one of the printing processes applicable to replace conventional photolithography processes for special applications such as flexible electronics [12,13], polymer electronics [14,15] and organic electronics [16,17] when resolution is not a concern. The process for the spacer layer in Figure 1(a) played an important role because the sensitivity of this 3DS sensing patch depends on not only the lateral dimension mentioned above, but also the vertical spacing (spacer height, *e*) [7] as indicated in Equation (8). The process flow, which combined the photolithography and the inkjet printing process, is illustrated in Figure 4. The first half, which defined the interferometer in specific dimensions, was done by conventional photolithography and vacuum evaporation while the second half, which defined the spacer height for the interferometer cavity, was done with the inkjet printing system (Fujifilm Dimatix DMP-2831).

A 125 μm polyethylene terephthalate (PET, Toray, T60) template was chosen as the substrate with 1 μm photoresist (Clariant, AZ5214-E) coating and UV exposure (Ushio, USH-250D, 3 mW/cm^2^) for 30 s, and then developed in developer (Clariant, AZ400K) for 20 s. Twenty nm silver (Ag) and 150 nm silicon-dioxide (SiO_2_) were deposited on the substrate after photoresist developing by sequential electron-beam evaporation (using a homemade tool). Lift-off process was performed in acetone for 300 s to entirely remove the photoresist and Ag with SiO_2_ above the photoresist. The decision of using lift-off rather by using etching was to avoid the chemical attack on the polymeric PET substrate. The SiO_2_/Ag structure represents the interference area and the exposed PET represents the area for the following spacer process by inkjet printing with UV sensitive resin (Chemiseal, 5X681).

**Figure 5.**
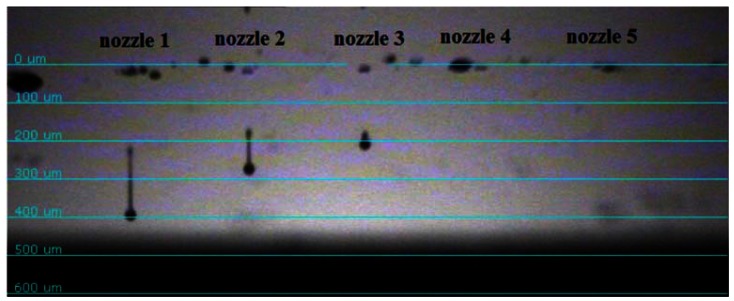
The dynamic droplet behaviors with tails and coffee ring concerns (nozzle 1–2), optimized condition (nozzle 3), and insufficient generation forces (nozzle 4–5).

Figure 5 showed the optimized dynamic droplet control to minimize the coffee ring effect [18,19] and the satellite effect [20] on the substrate by the inkjet printing system. The dynamic pictures were taken by high-speed camera with simulated fine droplet movement resolution of 1 μs/shot. Generally speaking, higher piezoelectric voltage provided longer droplet tail which in turn enhanced an unexpected satellite effect and lower piezoelectric voltage showed potential concerns of it not being possible to initiate droplets. The contact angle between the droplet and the substrate, droplet size, and droplet space of the optimized droplet was 9.2°, 70 μm, and 50 μm, respectively. The lower substrate was prepared similarly to the upper substrate with inkjet parameters mentioned above for the formation of spacers at the corners as shown in Figure 4(e). After the preparation of the inkjet process, the two layers were aligned and laminated with homemade stages under a microscope. A final UV curing process was applied on the whole structure for resin solidification and layer lamination. The spacer height of two samples after UV curing (100 mW/cm^2^ for 120 s) was 3 μm for sensitivity analysis.

## 4. Result

**Figure 6.**
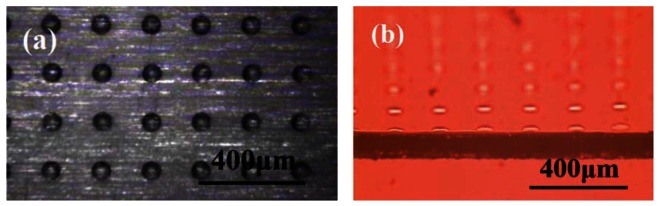
The optical microscope pictures of the test droplet array from (**a**) top and (**b**) cross sectional view.

**Figure 7.**
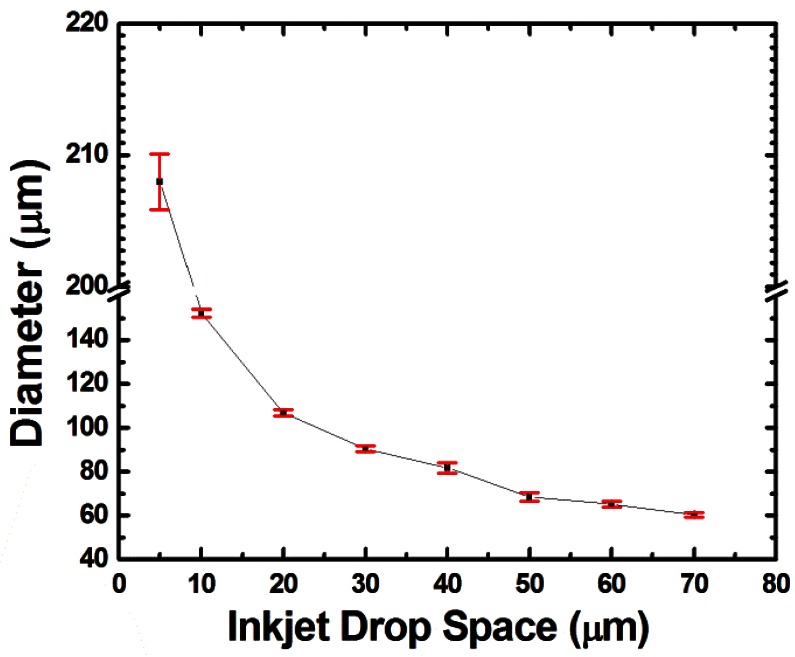
The relationship between the droplet space and the inkjet printed size (diameter).

The optimized static droplet array is shown in Figure 6, where the individual droplets had good shape and spacing without satellite effects. Each droplet had a volume of 10 pL. The lamination force between two layers depended on the droplet size according to the relationship between size and space shown in Figure 7. During lamination, the droplets expanded and this resulted in a greater lamination area. After curing, the solidified UV resin provided sufficient bonding force for buckling tests. The UV resin provided for an easier bonding process on a nanometer thickness scale compared to other studies, in which additional complicated processes such as plasma treatment [21], chemical treatment [22], and thermal reflow [23] were employed. Compared to the work done with gravure printing [7], this inkjet printing process provided controllable layer thickness and the possibility of precise positioning.

**Figure 8.**
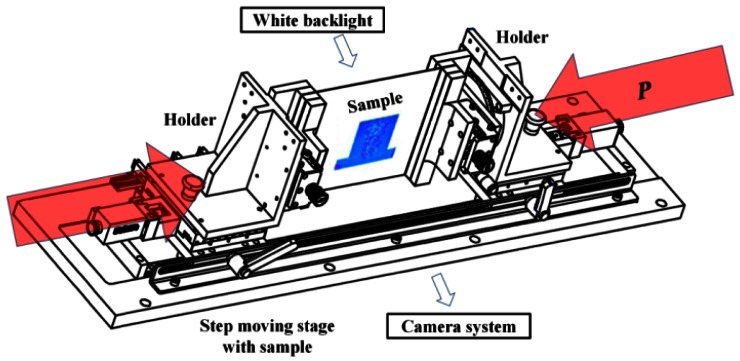
The setup of the step moving apparatus. The sample was placed between two holders and suffered balanced load from each side. The white backlight and the camera system were located on the opposite sides of the apparatus for transmissive image sensing.

**Figure 9.**
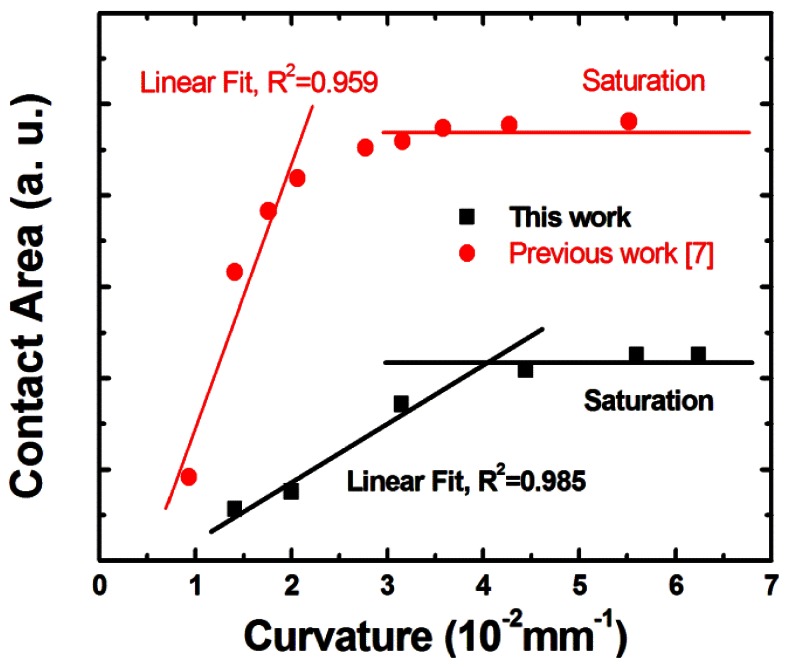
The expression of the sensitivity of the 3DS sensing patch. A saturation followed the linear region.

Figure 8 illustrates the newly designed apparatus for the buckling test. The sample was placed freely on the holder and was supported by itself without initial bending by gravity. When the two holders moved towards each other, the sample received equal loading (*P*) from both sides and buckled following the prediction of the model. A white backlight and a camera system were placed on the opposite sides of the apparatus for image capturing. The white backlight was the light source and the observed color changed to the interfered ones when buckling and contact occurred. The camera took pictures simultaneously along the step moving of the holders. The pictures were then fed to an image analyzing system where the interference (if any) contrast was enhanced, the contour was taken, and the contact area size was calculated. The movement of the apparatus was 5 μm/s and the sampling rate for pictures was 1 fps. Pictures with large contact area differences for better recognition were selected randomly for data collection. The sample (20 mm for *D*) with proper drop space and the optimized spacer height showed linear sensitivity in Figure 9 until it reached saturation. This 3DS sensing patch demonstrator supported a largest and a smallest bending radius of 70.72 mm and 16.02 mm in the linear region, respectively.

The buckling model introduced the spacer height (*e*) which influenced the requirement of the maximum displacement difference (Δ*v*_max_) as described in Equation (8). The maximum displacement of both the layers in turn influenced the contact area size. Compared to previous work done by gravure printing [7] with thinner spacer (*e* = 600 nm), this work (*e* = 3 μm) showed a reasonable curvature sensitivity (slope) trend, which satisfied the prediction of the model. The similarity between previous and this work was proved from the interpolation of both data sets listed on Table 2.

**Figure 10.**
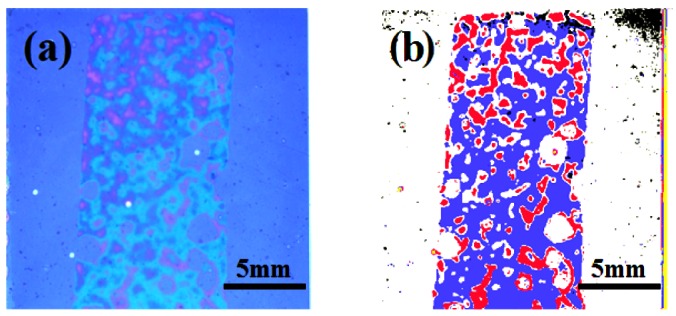
The image recognition steps of (**a**) initial and (**b**) contrast enhanced pictures.

Figure 10 shows the 3DS sensing patch in operation and its cropped image. When monitoring, the interference color was neglected because the optical path length difference of the interference lights slightly changed under buckling state. Thus, the contact areas were all counted no matter what their colors were. The colors appearing in the original picture (Figure 10(a)) was separately enhanced (Figure 10(b)) in different colors before they were counted for other studies, although only the outermost contour was taken into consideration in this work. The background blue color in Figure 10(a) was enhanced and modified to appear as white in Figure 10(b). The cyan and navy blue areas in Figure 10(a) were the color interference results, which were enhanced and modified so they appeared as blue in Figure 10(b). The violet purple areas in Figure 10(a) were also the color interference results, which were enhanced and modified to appear as red in Figure 10(b).

Because of the environmental limit for printing process, unexpected particles and air bubbles appeared during lamination, which did not influence the determination of the outermost contour [7]. The image analyzing program further took linear fit for the outermost pixels for boundaries of the contact area. Repeated reliability tests showed small, reasonable, and reliable data variation.

## 5. Conclusions

This work accomplished several novel ideas. Firstly, the buckling model was successfully built with mathematical calculations and simulation results. Not only did the buckling model support the simulation, but the experimental results also validated the buckling model. Secondly, an inkjet printing process was introduced to the manufacturing system which contained conventional photolithography steps. The precise control of inkjet droplet jetting, size, contact angle, and space, provided a controllability of the spacer height that previously was impossible. A high sensitivity result was demonstrated and extra options for different operation considerations can be expected. Thirdly, the image analyzing system made the whole process completely automatic. The program showed reliable data by enhancing the contrast of the interference area, taking its contour, fitting the outermost pixels and neglecting defects embedded inside, and calculating the size of the interference area.

This system took advantage of white backlight for transmissive color interference. The white backlight could be natural or artificial and the interference style could also be designed for a reflective one [24], which shows application on non-transparent object. An attractive application idea can be the monitoring system for large area flat panel display industry such as glass. Currently the glass industry uses lasers to monitor glass's flatness, which takes time when the area becomes larger. The laser system itself also suffers from serious influences from environmental fluctuation, let alone any physical vibrations or indexes of refraction changes. This 3DS sensing patch does not require a power supply and artificial light source, and thus may find a special niche in industry applications.

Compared to the buckling methodologies of other flexible electronic devices [25,26], the novel buckling system presented in this work provided precise control and tuning capability on curvature by adjusting the droplet size (Figure 7), which in turn changes the spacer height and the sensitivity. This proved that the accommodation of inkjet printing process successfully demonstrated comparable results and provided more options such as the spacer height, location control, and easy lamination process that could not be implemented by photolithography techniques.

## Figures and Tables

**Table 1.** Parameters used in this work.

*v*	Displacement in *z*-direction
*x*	Distance from device edge in *y*-direction
*D*	Device width
*F*	Axial loading in *y*-direction
*P*	Distributed loading in *y*-direction
*e*	Spacer height
*E*	Young's modulus of the substrate
*I*	Moment of inertia of the structure
*t*	Substrate thickness
*d*	Eccentric distance between the centroid of the structure cross section and the axial loading
*N*	Normal force

**Table 2.** Data points for previous work [7] and this work.

**Previous Work** [7]		**This Work**	

**Move Distance (*x*, mm)**	**Curvature (×10^−2^ mm^−1^)**	**Move Distance (*x*, mm)**	**Curvature (×10^−2^ mm^−1^)**

0.155	0.93	0.05	1.41
0.255	1.20	0.1	2.00
0.355	1.41	0.25	3.15
0.455	1.60	0.5	4.44
0.555	1.77	0.8	5.60
0.655	1.92	1.0	6.24
